# A review of graphene derivative enhancers for perovskite solar cells

**DOI:** 10.1039/d1na00830g

**Published:** 2022-03-22

**Authors:** Edwin T. Mombeshora, Edigar Muchuweni, Rodrigo Garcia-Rodriguez, Matthew L. Davies, Vincent O. Nyamori, Bice S. Martincigh

**Affiliations:** School of Chemistry and Physics, University of KwaZulu-Natal Westville Campus, Private Bag X54001 Durban 4000 South Africa martinci@ukzn.ac.za; SPECIFIC IKC, Materials Science and Engineering, Faculty of Science and Engineering, Swansea University Swansea UK M.L.Davies@swansea.ac.uk

## Abstract

Due to the finite nature, health and environmental hazards currently associated with the use of fossil energy resources, there is a global drive to hasten the development and deployment of renewable energy technologies. One such area encompasses perovskite solar cells (PSCs) that have shown photoconversion efficiencies (PCE) comparable to silicon-based photovoltaics, but their commercialisation has been set back by short-term stability and toxicity issues, among others. A tremendous potential to overcome these drawbacks is presented by the emerging applications of graphene derivative-based materials in PSCs as substitutes or components, composites with other functional materials, and enhancers of charge transport, blocking action, exciton dissociation, substrate coverage, sensitisation and stabilisation. This review aims to illustrate how these highly capable carbon-based materials can advance PSCs by critically outlining and discussing their current applications and strategically identifying prospective research avenues. The reviewed works show that graphene derivatives have great potential in boosting the performance and stability of PSCs through morphological modifications and compositional engineering. This can drive the sustainability and commercial viability aspects of PSCs.

## Abstract 2

This review discusses how incorporating graphene derivatives as components of perovskite, charge transport and blocking layers of perovskite solar cells affects substrate coverage, defect passivation, morphology and charge extraction/transport.

## Graphical abstract

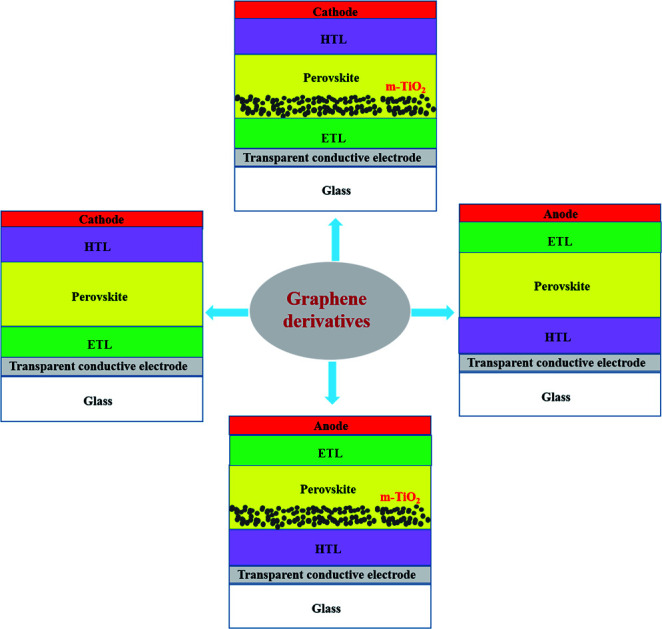

## 1. Introduction

As the global village advances in machinery and technology that sustains and enables humankind to live comfortably, environmental and health issues are also rising from the extensive dependency on highly polluting fossil-based energy resources. Fossil resources currently provide 81% of the global total energy supply.^1^ Besides their contribution to climate change, the combustion of fossil-based energy resources is also causing hazardous effects on the health of the world's population. For example, 18% of the total global deaths in 2018 were from pollution-related ailments emanating from the use of fossil-based energy resources.^2^ According to 2018 statistics from the World Health Organization (WHO), the use of fossil-based energy resources has mostly affected children (>40% environmental-related diseases and >88% of the health complications from climate change were experienced by children under five years).^3^ Economic and health costs (work absenteeism, lives lost and premature deaths) due to air pollution from burning fossil fuel energy resources ascended to $2.9 trillion in 2018.^4^ Since fossil-based energy resources are non-renewable and causing alarming damaging outcomes, other alternatives need to be developed to dependable levels.

One hugely important renewable energy technology is photovoltaics, with a global energy output growth of 60% between 2000 and 2016.^5^ For instance, in Europe, where 70% of the global total installed solar cells are located, annual PV installation rose from 58 MW to 222 GW between 2000 and 2015.^6^ In this ambit, perovskite solar cells (PSCs) in particular, are not only a rising energy technology but a cheaper option as well. In 2016 the hypothetical levelized cost of electricity (LCOE) for a perovskite solar cell (PSC) with PCE of 20% and a projected PSC life span > 15 years was calculated to be 3.5 US cents per kW h, whilst the LCOE for traditional energy resources, such as oil and gas, was in the range US 7.04–11.90 cents per kW h.^7,8^ PSCs have emerged with great potential to compliment market-leading Si PV due to their high PCE and potentially low manufacturing costs.^8,9^ Additionally, in 2017, Song *et al.*^10^ stated that the production cost of a standard PSC was US $31.70 m^−2^ ($6.80 m^−2^ was PSC processing costs and $24.90 m^−2^ was the balance of module (BOM) components). This means the manufacturing cost of PSCs, after excluding BOM component expenditure, is lower than that of other thin-film solar cells (CIGS: US $29 m^−2^ and CdTe: US $27 m^−2^).^8,10^ This analysis also infers that the major contributor to PSC costs is the BOM components.

Additives and substitution of BOM components with lower-cost alternatives is a promising approach in balancing the possible trade-off between cost and PCE. In a PSC, 76% of the total expenditure is from materials; however, the cost of the material in the perovskite unit cell structure and active material contributes only 7.6% to this.^10,11^ Examples of expensive BOM components in PSCs include noble metals, such as Au, conductive substrates, and hole transporting layers (HTLs), such as spiro-OMeTAD.^12,13^ The use of lower-cost, alternative sustainable materials or strategies (such as remanufacturing) will be crucial in minimising LCOE and improving the chance of commercial success.

Although solar cells have the potential to meet clean global energy demands, the waste from photovoltaics after reaching their end of life is rising to alarming levels. For example, the cumulative photovoltaic waste is projected to reach 78 million tonnes by 2050.^6^ One strategy to help alleviate this problem is the greater use of eco-friendly materials that can reduce both bio-incompatible waste accumulation and recycling costs. Coupled with this waste problem is the growing use of primary materials, with renewable energy technologies requiring significant amounts of critical raw materials. To ensure rapid deployment of renewable technologies is not disrupted, materials with inherent natural abundance are needed. Graphene derivatives are one such class of materials as they can be obtained from the abundant and naturally occurring graphite and biomass. To date, sustainable additives such as graphene derivatives have principally shown potential in aiding exciton dissociation, conductivity and long-term stability in PSCs (reviewed articles in Table 1).

**Table 1:** Illustrative applications of graphene derivatives in perovskite solar cells^a^

Device structure		Stability			*J*_sc_ (mA cm^−2^)	*V*_oc_ (V)	FF (%)	Champion PCE (%)	Ref.
		Conditions^a^	Time (d)	PCE decline (%)					
**Spin-coating deposition of graphene-based materials**									
**As HTL component and electron blocking layer in p–i–n configuration**									
Glass/ITO/GO/PEDOT:PSS/MAPbI_3_/PCBM/rhodamine 101/LiF/Au		n/d	n/d	n/d	18.20	0.97	80.00	14.10	82
Glass/ITO/GO/PEDOT:PSS/MAPbI_3_/PCBM/carbon tape		Ambient	4	0	13.80	0.80	48.00	5.20	193
Glass/Ag nanowire–GO/PEDOT:PSS/MAPbI_3_/PCBM/2,9-dimethyl-4,7-diphenyl-1,10-phenanthroline (BCP)		n/d	1	0	15.43	0.87	70.90	9.62	122
Glass/ITO/PEDOT:PSS–GO/(FAPbI_3_)_0.85_(MAPbBr_3_)_0.15_/PCBM/BCP/Ag		n/d	n/d	n/d	20.01	0.90	79.00	14.20	108
Glass/ITO/PEDOT:PSS–GO/MAPbI_3_/PC_70_BM/Al		n/d	n/d	n/d	17.92	1.03	71.00	12.76	162
Glass/ITO/PEDOT:PSS–NH_3_–GO/MAPbI_3_/PCBM/Bphen solution/Ag		Air	4	28	22.06	1.03	71.00	16.11	85
Glass/ITO/PEDOT:PSS–PANI–GO/MAPbI_3_/PCBM/rhodamine 101/Ag		Ambient, RH: 20%	80	70	22.89	1.05	75.40	18.12	94
Glass/FTO/PANI–GO/MAPbI_3_/PCBM/Ag		n/d	n/d	n/d	21.23	0.52	67.00	9.24	160
Glass/ITO/PEDOT:PSS–RGO/MAPbI_3_/PCBM/BCP/Ag		n/d	n/d	n/d	16.75	0.87	75.00	10.70	194
Glass/ITO/PEDOT:PSS–RGO/MAPbI_3_/PCBM/Al		n/d	n/d	n/d	17.10	0.95	65.00	10.60	195
Glass/ITO/PEDOT:PSS–sulfur–RGO/MAPbI_3_/PCBM/Ag		n/d	n/d	n/d	19.40	1.01	67.00	13.00	196
Glass/ITO/PEDOT:PSS–sulfonic acid–RGO/MAPbI_3_/PCBM/BCP/Ag		Ambient air	30	12	19.39	1.04	80.48	16.01	117
Glass/ITO/PEDOT:PSS/poly(ethylene oxide)–GO/MAPbI_2.5_Br_0.5_/PCBM–MoS_2_/Ag		AM: 1.5 G	17	6	22.83	1.14	73.80	19.14	197
Glass/FTO/NiO/GO/MAPbI_3−*x*_Cl_*x*_/GO–Li/TiO_*x*_/Al		Ambient air, RH: 20–38%	15	30	18.60	0.97	62.00	11.20	168
Glass/ITO/PEDOT:PSS/fluorinated RGO/MAPbI_3_/PCBM/BCP/Ag		n/d	n/d	n/d	19.10	1.01	76.2	14.70	51
Glass/ITO/PEDOT:PSS/MAPbI_3_/PCBM–RGO/poly[(9,9-bis(3′-(*N*,*N*-dimethylamino)propyl)-2,7-fluorene)-*alt*-2,7-(9,9-dioctylfluorene) (PFN)/Ag		Continuous light, RH: >50	5	45	22.92	0.85	65.80	12.82	198

**Solely as HTL in p–i–n configuration**									
Glass/ITO/GO/MAPbI_3−*x*_Cl_*x*_/PCBM/ZnO/Al	n/d		n/d	n/d	17.46	1.00	71.00	12.40	25
Glass/ITO/GO/FA_0.2_MA_0.8_Pb(I_0.8_Br_0.2_)_3_/PCBM/ZnO/Ag	RH: 65–75		4	60	21.00	1.00	71.00	14.90	118
Glass/ITO/GO/C quantum dots/MAPbI_3_/PCBM/BCP/Ag	Temperature (*T*): 25 °C RH: 25–30%		2	10	18.70	0.95	80.10	16.20	199
Glass/FTO/GO/MAPbI_*x*_Cl_3−*x*_/TiO_2_–Li–GO/Al	n/d		n/d	n/d	15.60	0.91	72.00	10.20	115
Glass/FTO/N–GO nanoribbons/MAPbI_3_/ZnO/Al	*T*: 20 °C, RH: 47%		2	11	17.42	1.00	71.30	12.41	200
Glass/FTO/NH_3_–GO/MAPbI_3−*x*_Cl_*x*_/PCBM/bathocuproine (BCP)/Ag	n/d		30	10	18.40	1.00	76.80	14.14	158
Glass/ITO/RGO/MAPbI_3_/PCBM/Ag	*T*: 25 °C, RH: 30%, no encapsulation		41	50	22.1	0.96	77.00	16.40	201
	Bending cycles (cycles): 150		n/d	n/d	30.00	n/d	n/d	n/d	
Glass/ITO/RGO/Cu(i) thiocyanate (CuSCN)/MAPbI_3_/PCBM/BCP/Ag	Continuous AM = 1.5 sun		4	10	18.21	1.03	76.10	14.28	202
Glass/ITO/MoO_3_–RGO/MAPbI_3_/PCBM/PCP/Ag	Encapsulated, RH: 30%		30	35	21.18	1.12	77.00	18.15	177
Glass/ITO/poly-(*N*-vinyl pyrrolidone)–RGO/MAPbI_3_/PCBM	n/d		42	50	14.86	0.97	79.95	11.36	203

**In composite with ETL and as a hole blocking layer in n–i–p configuration**									
Glass/ITO/ZnO–GO/MAPbI_3_/Au		n/d	n/d	n/d	21.51	0.67	54.00	4.52	204
Glass/ITO/compact TiO_2_ (c-TiO_2_)/m-TiO_2_/Li–GO/MAPbI_3_/spiro-OMeTAD/Au		1 sun	2.5	17	19.61	0.86	70.30	11.14	120
Glass/ITO/SnO_2_–N–GO/MAPbBr_3_/spiro-OMeTAD/Au		Ambient air, *T*: room, RH: 25	n/d	12	18.87	1.17	74.93	16.50	35
Glass/FTO/c-TiO_2_/m-TiO_2_–GO/mesoporous-ZrO_2_/MAPbI_3_/carbon		n/d	n/d	n/d	22.84	0.98	61.72	13.60	205
Glass/FTO/c-TiO_2_/m-TiO_2_–RGO/MAPbI_3−*x*_Cl_*x*_/Cu–Bu–phthalocyanine		Ambient air, *T*: room, RH: 28–32%	n/d	34	21.00	1.07	71.00	15.90	27
Glass/FTO/c-TiO_2_/m-TiO_2_–RGO/MAPbI_3_/spiro-OMeTAD–Li		n/d	n/d	n/d	22.00	0.93	70.70	14.50	26
Glass/FTO/c-TiO_2_/m-TiO_2_–PANI–RGO/CsPbI_3_–PbI_2_/spiro-OMeTAD/Au		Encapsulated, *T*: 20–30 °C, RH: 20%	77.9	18	26.96	0.96	63.60	16.48	206
Glass/FTO/Bl–TiO_2_/rGO_4_–TiO_2_/(FAPbI_3_)_0.85_(MAPbBr_3_)_0.15_/spiro-OMeTAD/Au		n/d	n/d	n/d	22.16	1.07	75.40	17.66	207

**As a constituent of the active layer in n–i–p configuration**									
Glass/ITO/SnO_2_/MAPbI_3_–GO/spiro-OMeTAD/Au		n/d	n/d	n/d	23.73	1.07	69.14	17.59	208
Glass/FTO/SnO_*x*_/Cs_0.05_(FA_0.85_MA_0.15_)_0.95_Pb(I_0.85_Br_0.15_)_3_–dodecylamine–GO/spiro-OMeTAD/Au		Room temperature, no encapsulation	40	30	22.10	1.10	81.00	21.10	106
Glass/FTO/TiO_2_/MAPbI_3_–CNT–P3HT–GO/spiro-OMeTAD/MoO_3_/Au		n/d	n/d	n/d	22.73	0.96	75.00	16.36	93
Glass/FTO/c-TiO_2_–m-TiO_2_/FA_0.8_MA_0_._16_Cs_0.04_Pb(I_0.84_Br_0.16_)_3_–RGO/spiro-OMeTAD/Au		*T*: 85 °C, RH: 40%	2.5	20	24.00	1.15	76.00	19.34	46
Glass/FTO/TiO_2_–RGO/TiO_2_/MAPbI_3_–RGO/spiro-OMeTAD/Ag		Mild humid, dark	50	60	22.90	1.01	72.00	16.50	107
Glass/FTO/c-TiO_2_/m-TiO_2_/Al_2_O_3_/MAPbI_*x*_Cl_3−*x*_–Ag–RGO/spiro-OMeTAD/Au		*T*: 25–30 °C, RH: 45–57%	330	0	22.80	n/d	n/d	n/d	103
Glass/FTO/c-TiO_2_–RGO/m-TiO_2_–MAPbI_3_–RGO/spiro-OMeTAD/Au		n/d	n/d	n/d	16.50	0.84	58.30	9.30	34
Glass/FTO/c-TiO_2_/m-TiO_2_–MAPbI_3−*x*_Cl_*x*_–RGO/spiro-OMeTAD/Au		n/d	n/d	n/d	22.30	0.93	74.00	15.30	209
Glass/FTO/c-TiO_2_/m-TiO_2_/MAPbI_3−*x*_Cl_*x*_–N–RGO/spiro-OMeTAD/Au		n/d	n/d	n/d	21.80	1.15	74.00	18.73	138
Glass/FTO/c-TiO_2_/(FAPbI_3_)_0.85_(MAPbBr_3_)_0.15_–c-TiO_2_–Li–RGO/spiro-OMeTAD/Au		n/d	n/d	n/d	21.98	1.11	80.00	19.54	124

**As HTL and electron blocking layer in n–i–p configuration**									
Glass/FTO/TiO_2_/MAPbI_3_/GO/Au		Dark, no encapsulation	30	50	8.00	0.80	51.25	3.28	210
Glass/FTO/c-TiO_2_/m-TiO_2_–graphene/MAPbI_3_/GO/spiro-OMeTAD/Au		1 sun	0.67	67	22.48	1.08	75.12	18.19	211
Glass/FTO/c-TiO_2_/m-TiO_2_–MAPbI_3−*x*_Cl_*x*_/GO/Cu–Bu–phthalocyanine/Au		n/d	n/d	n/d	20.90	1.04	66.00	14.40	212
Glass/FTO/c-TiO_2_/m-TiO_2_/CsPbBr_3_/polyvinyl acetate/GO/carbon		*T*: 25 °C, RH: 80%, no encapsulation	29	3	7.41	1.55	82.80	9.53	13
Glass/FTO/c-TiO_2_/m-TiO_2_/MAPbI_3−*x*_Cl_*x*_/amine–GO/P3HT/Au		n/d	n/d	n/d	24.43	0.93	58.00	13.25	144
Glass/FTO/TiO_2_/MAPbI_3_/RGO/spiro-OMeTAD/Au		Air	20	15	16.73	0.91	61.00	10.60	154
Glass/FTO/Sr–TiO_2_/Al_2_O_3_–graphene/NiO/MAPbI_3−*x*_Cl_*x*_/NiO–RGO/spiro-OMeTAD/Au		*T*: 25 °C	310	3	25.90	1.05	76.40	20.80	111

**In composites with HTL in n–i–p configuration**									
Glass/FTO/SnO_2_/FAMAI_3−*x*_Br_*x*_/spiro-OMeTAD–RGO/Au		Ambient	21	25	23.05	1.10	71.00	18.13	213
Glass/FTO/c-TiO_2_/m-TiO_2_/MAPbI_3_/spiro-OMeTAD–poly(methyl)methacrylate–RGO/Au		*T*: 35 °C, RH: 40%	42	7	22.60	1.01	68.00	15.70	214
Glass/FTO/C_60_/MAPbI_3_/spiro-OMeTAD/Li–TFSI/P3HT–4-(hexyloxy)phenyl)–RGO		n/d	n/d	n/d	20.00	0.87	55.00	10.00	215

**Lamination deposition of graphene derivative-based materials**									
Glass/GO/MoO_3_–PEDOT:PSS/MAPbI_3_/C_60_–BCP/LiF–Al		n/d	n/d	n/d	21.90	1.03	72.00	17.10	119
Glass/ITO/PEDOT:PSS–silver trifluoromethanesulfonate–GO/MAPbI_3−*x*_Cl_*x*_/PCBM/Au		n/d	n/d	n/d	19.18	0.88	70.51	11.90	116
Glass/FTO/TiO_2_/MAPbI_3_/B–RGO/FTO		Ambient, dry box, room light, RH: 60	10	n/d	16.74	0.88	60.00	8.96	159

**Electrospray deposition of GO layer**									
Glass/ITO/TiO_2_/MAPbI_3−*x*_Cl_*x*_/4-fluorophenyl-hydrazine hydrochloride–GO/spiro-OMeTAD/Au		RH: >50%, N_2_-filled glove box, 1 sun	5	70	21.50	1.11	78.60	18.80	152 and 216

**Spray deposition of RGO layer**									
Glass/FTO/c-TiO_2_/nano-crystalline-TiO_2_–MAPbI_3_/RGO/Au		n/d	n/d	n/d	11.50	0.95	60.54	6.62	217

**Radio-frequency magnetron sputtering GO deposition**									
Glass/ITO/GO/MAPbI_3_/Ag		n/d	n/d	n/d	7.80	0.92	24.43	1.80	128

**Unspecified deposition methods for graphene-based materials**									
Glass/FTO/GO/MAPbI_3_/PCBM/ZnO/Al		Ambient, *T*: <30 °C	20	20	18.06	1.10	77.70	15.20	218
Glass/bis(trifluoromethanesufonyl-amide–GO/PEDOT:PSS/FAPbI_3−*x*_Br_*x*_/PCBM		*T*: 60 °C, RH: 30%, 1 sun	42	5	22.70	1.07	77.70	18.90	219
Glass/ITO/RGO/MAPbI_3_/PCBM/BCP/Ag		Ambient	6	38	15.40	0.98	71.60	10.80	220
Glass/FTO/Zn–RGO/MAPbI_3_/spiro-OMeTAD/Au		n/d	30	10	21.70	1.03	68.00	15.20	178
Glass/FTO/spiro-bifluorene/MAPbI_3_–GO/PCBM/bathocuproine/Au		Ambient	30	4	18.80	1.07	71.00	14.28	29
Glass/FTO/TiO_2_/MAPbI_3_ bilayer/GO		n/d	n/d	n/d	16.70	0.94	73.00	11.50	171

a n/d – no details, RH – relative humidity, AM – air mass.

This review focuses on the application and suitability of graphene-based derivative additives as components of the substrate, active-, charge transport- and blocking-layers in PSCs. We pay particular attention to their influence on the stability and performance of PSCs, and reflect on any drawbacks these graphene derivative materials may pose when incorporated in PSCs.

## 2. Perovskite solar cells

Perovskites have the general formula ABX_3_, where A is the larger cation, typically CH_3_NH_3_^+^ (MA^+^), HC(NH_2_)_2_^+^ (FA^+^), Cs^+^ or mixed cations, such as FAMA, which occupy the cubo-octahedral site shared with twelve halide anions X^−^. B is the smaller cation (Pb^2+^, Sn^2+^, *etc.*) which is stabilised by the octahedral setup and shared with six halide anions, X^−^.^14–20^ An organic–inorganic hybrid perovskite is usually used in solar cells, and the methylammonium lead iodide (MAPbI_3_) is the most common (Table 1). Perovskites have a direct optical band gap of typically 1.5 eV, although this can be tuned through the visible region by altering their chemical compositions.^21–23^ The impressive performance of perovskites in solar cells is generally due to the high absorption coefficient (>10^4^ cm^−1^), long charge-carrier lifetime and long charge-diffusion path (>1 μm).^24–29^ A further advantage is the compatibility with low-cost solution processing techniques. In addition to quality (morphology and uniformity), the film thickness of the perovskite layer is an important factor for maximising the efficiency of devices. The high absorption coefficient of perovskites results in thin-films of *ca.* 400 nm being suitable for efficient photon capture. This is advantageous because it results in less use of materials; even though perovskites are typically composed of earth-abundant materials, this still benefits cost and helps reduce materials wastage.

**Fig. 1:**
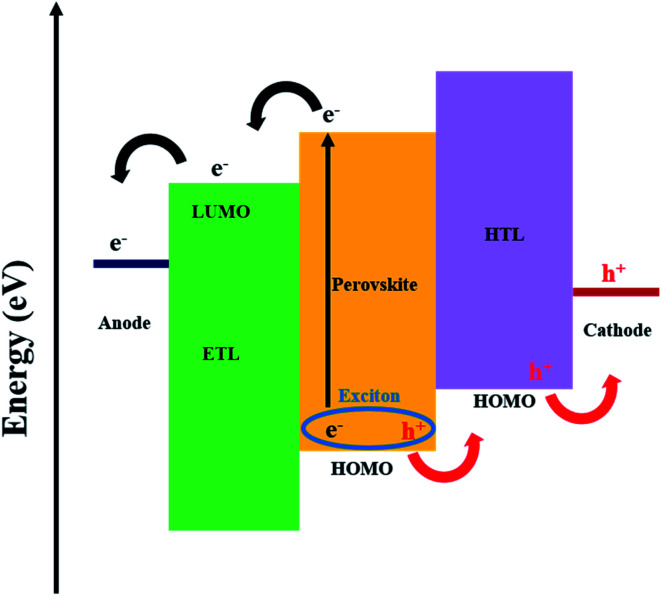
Schematic energy level diagram of a conventional PSC.

The working mechanism of a PSC involves the absorption of light by the perovskite layer; upon excitation, excitons quickly dissociate at room temperature due to a very low exciton binding energy, resulting in free charges. Electrons are extracted at the electron transport layer (ETL), while holes are extracted at the hole transport layer (HTL) prior to transportation to the anode and cathode, respectively (Fig. 1).^21^ Interfacial electron transfer from the perovskite conduction band to the HTL and/or ETL surface states, together with interfacial electron transfer from the conduction band of the ETL to the HTL and/or perovskite, are possible recombination mechanisms in PSCs (Fig. 1).^30^ The current review will, in later sections, discuss the potential of graphene derivatives to retard recombination in PSCs.

The PCE of PSCs has dramatically increased from about 10% in 2012 ^31^ to a certified value of 25.5% by 2019,^32,33^ which is comparable to the 26% PCE of monocrystalline Si-based solar cells.^33–43^ PSCs are well-positioned to be successfully commercialised since their PCE has surpassed other thin film-based solar cells (CIGS: 23% and CdTe: 22%).^33,44,45^ This significant improvement of PCE within the last decade is mainly attributed to morphological tailoring and compositional engineering of the PSC device layers.^46^ Relative to the first two photovoltaic generations, PSCs have the potential to harness light at lower costs and are also associated with facile fabrication procedures.^47^

Over 12 000 articles on PSCs had been reported by 2019, and since then, companies have been focusing on the commercialisation of the technology.^19,40^ PSCs have a great potential in finding a niche in the world energy markets because they are highly efficient, printable, can be noble metal- and HTL-free.^48^ For example, PSCs have high potential in wearable devices due to the associated facile fabrication, high PCE and low costs.^39,49,50^ To date, the technology has already advanced to mini- and standard-sized modules.^19^ For instance, Yeo *et al.*^51^ reported PCEs in the 8.1–10% range for scalable flexible and rigid modules with an area of 10 cm^2^. However, PSCs are still not found substantially in the global markets because the technology is in the laboratory/industrial scale transition phase.^21,48,52^ Roll-to-roll technology is more practical for optimum substrate coverage at large-scale due to low fabrication costs, sample nature, device structure and manufacturing steps involved. The transformation from laboratory to large-scale roll-to-roll deposition still needs more insights on several aspects, such as solvent selection, annealing temperature, and film thickness control and deposition rate, to avoid a substantial decrease in PCE.^53,54^

Additionally, the commercialisation and large-scale production of PSCs is also hindered by toxicity issues due to the use of solvents,^43^ such as DMF,^43^ γ-butyrolactone (GBL),^55^ chlorobenzene and toluene,^12,19^ and Ag and Pb as counter electrodes.^21,42^ Notwithstanding the fact that the toxicity of Pb(ii) salts is a problem, studies are yet to provide a lucrative prospective substitute material to Pb-based PSCs.^56^ Possible substitutes being considered include elements in the same periodic group, Sn and Ge; however, their toxicity, stability and band gap still need tailoring to achieve comparable absorption and PCEs.^15,57^ There is also the possibility of solvent-free approaches^58^ although control over uniformity and morphology can prove difficult.

Another key aspect hindering commercialisation is the stability of the perovskite layer with, to date, the best-reported device lifetimes of approximately one year.^14,28,59,60^ The reviewed research has shown interesting stability profiles of PSCs in terms of shelf-life, continuous illumination, short circuit (increased accumulation of charge), bending cycles and defined relative humidity (RH) (Table 1). In-depth degradation studies have shown that ageing effects on PSCs are not homogeneous.^61^ Additionally, other comprehensive studies have monitored the degradation profiles of PSCs under operating conditions. They infer that stability is centred on phase separation (in hybrid halides), interface state(s), photosensitivity, and thermal, oxygen and moisture effects that cause perovskite degradation.^9,61–66^ Moisture (RH of >55%) catalyses perovskite degradation to a polar state *via* H^+^ extraction from the perovskite and facilitates the dissolution of water-soluble components as MAI, causing a colour change.^15,67–69^ Oxygen detrimental effects occur when the molecules diffuse into the perovskite, and become trapped in the halide vacancies where interaction with an excited state forms reactive superoxide species.^15,62,64,69^ Ultraviolet and visible irradiation can also cause device degradation through accelerated ion migration.^28,57,64,70–72^ The blue to the ultraviolet region of the solar spectrum also triggers the dissolution of the perovskite organic component.^73^ Perovskite degradation to PbI_2_ can be thermally induced altering the physicochemical properties, such as a reduced optical absorption due to an enlarged band gap.^15,63^ Perovskite phase separations (cause photo-inactive states and current blocking) and phase transformations, such as FA_0.9_Cs_0.1_PbI_3_ to FA_>0.9_Cs_<0.1_PbI_3_, deteriorate PCE.^63,64^ Additionally, studies have shown that I_2_ vapour does not only cause localised degradation but also induces decomposition of neighbouring perovskite regions during operational conditions.^74,75^ Interestingly, under illumination, I_2_ is more easily released from PbI_2_ than from perovskite,^74^ meaning that once some perovskite decomposes to PbI_2_ more problems are initiated.

In research laboratories, device fabrication is carried out in a glove box to ensure a controlled atmosphere, but this may not be easily achieved in large-scale industrial production. In addition, real-world PSCs will be applied in RH environments that exceed the laboratory average of 30–50%.^76^ With the current state of the art, real-world devices will need to be fabricated in such a way as to limit humidity and oxygen exposure. Thus, more work is needed to develop new charge selective carriers, and engineer new perovskite materials, interfacial modifications, and novel configurations to achieve long-term stability.^15^

### 2.1 PSC configurations and components

**Fig. 2:**
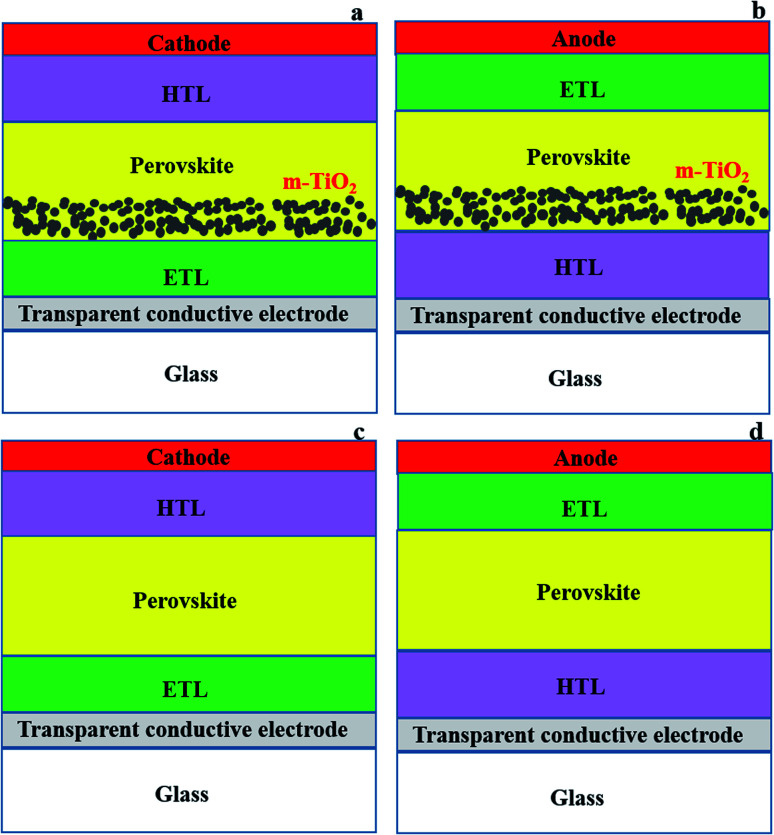
Mesoporous (a) n–i–p and (b) p–i–n, and planar (c) n–i–p and (d) p–i–n PSC configurations.

The basic architectures of PSCs are either mesoporous (Fig. 2a and b) or planar (Fig. 2c and d). Mesoporous devices have a mesoporous (often TiO_2_) layer on top of the compact ETL, whereas planar devices just have a compact ETL layer.^77–79^ Perovskites are suitable for planar structural solar cells due to their ability to transport holes and electrons simultaneously, and have long exciton diffusion lengths (100–1000 nm) and favourable excited-state lifetimes (100 ns).^56,80^ PSC devices can be manufactured in both n–i–p (transparent conducting electrode (TCE)/ETL/perovskite/HTL/cathode (Fig. 2a and c))^24,36,41^ and p–i–n (TCE/HTL/perovskite/ETL/anode (Fig. 2b and d)) configurations.^25,81,82^ The difference between n–i–p and p–i–n is simply the position of the ETL and HTL relative to the transparent substrate and counter electrode (Fig. 2).^28^

Studies on planar PSCs have highlighted the need for critical crystallisation and growth control to minimise morphological deviations (*i.e.*, defects; imperfect crystals and undesirable interfacial states) and recombination.^83,84^ The carrier lifetime, charge extraction and charge transfer issues that cause charge recombination and thus lower photocurrent in PSCs, particularly for planar architecture, have been linked to the ETL used.^44^ Other common issues related to the introduction of charge transport layers in PSCs include the cost of materials, deterioration of electronic characteristics due to poor alignment of energy levels and charge extraction (slightly higher conduction band of ETL and lower HOMO of HTL than the valency band of perovskite, respectively, is needed to allow charge transport), slow electron mobility, high hydrophilicity of components causing poor chemical stability, and short-term stability and poor perovskite quality (creation of void infested morphology, poor crystallisation and crystal boundary state).^21,85,86^

Generally, defects commonly found in perovskites can be categorised as intrinsic 0D (vacancy, interstitial and anti-site substitutions), 0D from impurities and 2D (surface defects, pinholes and grain boundaries).^59,87^ The perovskite grain boundaries are weaker than the interior for several reasons, such as the possible voids generated during precursor solidification, localised large volume and high stress intensity existence, and occurrence of amorphous boundary structures from Pb–X lattice distortions.^88^ The interstitial and substitutional defects are deep level imperfections that lower perovskite stability and create charge traps.^89^ Since perovskite film morphology affects stability and rate of degradation,^90^ the quality of perovskites has been improved by optimisation of preparation conditions and compositional engineering, such as the use of graphene additives and selecting suitable starting materials.^46,89,91,92^ Additionally, defect passivation can reduce non-radiative recombination and energy losses, and readers are referred to detailed reviews by Gao *et al.*^59^ and Zhang *et al.*^14^ on defect passivation. In brief, passivation is when a material's reactive/unstable component is made to become less affected, if not at all, by an external environment.^59,68,90^

Graphene derivatives have shown potential in improving substrate coverage, defect passivation, and charge extraction/transport (as highlighted in relevant sections and Table 1). For instance, the inclusion of graphene oxide (GO) in the perovskite layer of a PSC lowered series resistance (*R*_s_) from 27.8 to 15.2 Ω.^93^ This was ascribed to enhanced crystallinity of the perovskite in the composite. Additionally, surface potentials of a PSC were lowered with an increase of both *J*_sc_ and *V*_oc_ by lowering the HOMO level through the addition of polyaniline (PANI) to a GO/poly(3,4-ethylenedioxythiophene):polystyrene sulfonate (PEDOT:PSS) composite.^94^ Graphene derivatives have great potential in improving some of the most significant drawbacks of optimising PSCs. Despite this great potential, the use of graphene-based materials in PSCs is still at primitive stages;^42^ and, hence, needs constant review to reveal their potential holistically.

## 3. Application of graphene derivatives in perovskite solar cells

**Fig. 3:**
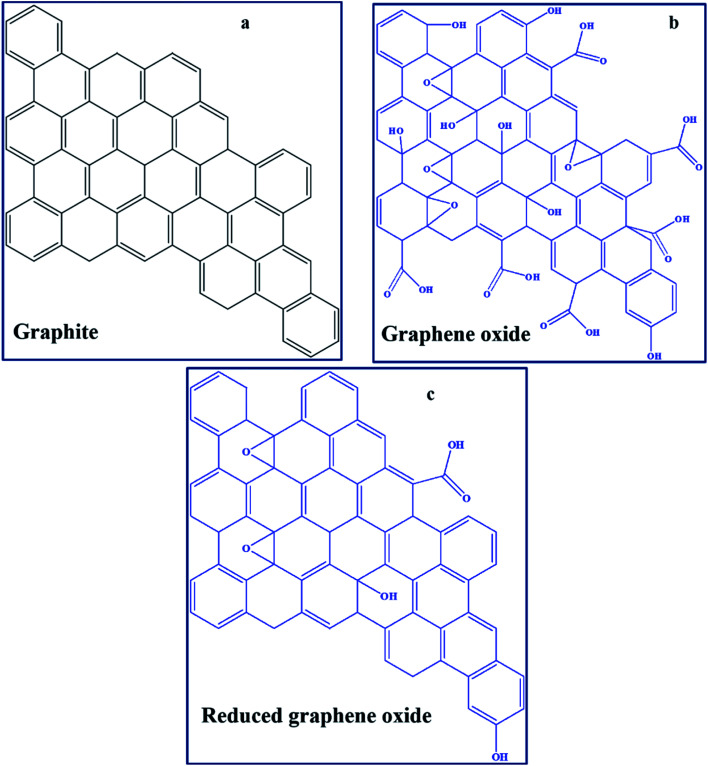
Basic chemical structures of (a) graphite, (b) GO and (c) RGO.

GO can be defined in simple terms as an oxide form of graphene that is chemically synthesised from graphite (Fig. 3a and b).^95,96^ Technically, the material obtained after partial removal of oxygen functionalities is termed reduced graphene oxide (RGO).^96^ Furthermore, the development of several graphene derivatives has generated numerous and sometimes confusing terms, such as transferred, suspended, epitaxial grown, chemical vapour deposition (CVD)-grown, isolated-freestanding, and nanoplatelets, among others,^97,98^ which represent GO-based materials with different physicochemical properties. The major graphene derivative examples applicable in PSCs include GO (Fig. 3b), RGO (Fig. 3c), nitrogen-doped GO/RGO (N–GO/–RGO), boron-doped GO/RGO (B–GO/–RGO) and metal oxide functionalised–GO/–RGO (Table 1). A high concentration of oxygen functionalities, particularly in GO, is usually associated with insulating characteristics, while a low oxygen concentration leads to high conductivity due to the restoration of the pi-system towards pure graphene. Hetero-atom doped graphene derivatives, such as those that are N-doped, also have enhanced conducting behaviours.

A comprehensive summary of the use of graphene derivatives in PSCs is presented in Table 1. It shows that there were few reports on graphene-based PSCs in the last decade, probably due to the drawbacks that generally evolve around high-temperature synthesis requirements.^21,47^ Graphene derivatives are mostly applied as components of the HTL, ETL, blocking layer, and conducting electrode and, rarely, in the active layer of PSCs. The PCE values of graphene-based PSCs have grown from about 6.6% to the current value of approximately 17%.^99^ Although the champion PCE of graphene-based PSCs is currently lower than the certified champion PCE value for PSCs (of 25.5%),^33^ the relative increase in performance of graphene derivative-based devices from their first use, has been attributed to several factors that are inclusive of chemical inertness, increased charge transfer pathways, and surface area. For instance, a relatively large surface area of 202 m^2^ g^−1^ in a graphene derivative-based PSC (graphene derivative applied on top of mesoporous ZrO_2_ and TiO_2_) created a high number of interconnected channels that allowed perovskite precursor penetration and decreased the photoluminescence (PL) lifetime (from 1.03 to 0.698 ns).^100^

The use of graphene grown by CVD in PSCs potentially introduces high costs for large-scale manufacturing because of complicated transfer steps of graphene onto the target substrate. In addition, it is associated with both poor contact and minimum film thickness control.^21^ Hence, GO is more appropriate for large-scale PSC development than graphene because solution processable GO synthesised *via* several chemical exfoliation methods^96^ can disperse well in several solvents compatible with PSC fabrication, and allows for potential further functionalisation.

Since the mechanism of PSCs is inclined to the charge transport layer used for quenching charge carriers,^25^ fast charge decay is affected by fast transport of free carriers from the perovskites to respective contacts.^101^ The electron and hole mobility need to be balanced in PSCs, and this can be attained through the synthesis of composites, such as polymer-perovskite,^102,103^ metal oxide-perovskite^104,105^ and graphene derivative-perovskite composites.^46,106^ For instance, GO-perovskite composites were reported to increase charge separation (PL coefficient due to non-radiative surface-state relaxation of perovskite was one-tenth of that of the composites), charge mobility (from 29 to 35 cm^2^ V^−1^ s^−1^) and recombination resistance (from 8865 to 99 978 Ω cm^2^).^80^ Also, the addition of RGO to mesoporous TiO_2_ [(m-TiO_2_)–RGO] was reported to double the electron diffusion coefficient from that of a m-TiO_2_ ETL.^26^ In another study, the electron mobility of m-TiO_2_ was raised from 2.5 × 10^−7^ to 4.2 × 10^−7^ cm^2^ V^−1^ s^−1^ in a RGO–m-TiO_2_ ETL.^107^ Doping of PEDOT:PSS with GO was reported to improve the hole mobility from 5.55 × 10^−5^ to 1.57 × 10^−4^ cm^2^ V^−1^ s^−1^.^108^ These studies demonstrate the potential of graphene derivatives in promoting charge mobility *via* the provision of additional pathways for effective excited-state charge carriers and charge transport away from the PSC active layer (Table 1). This is partly due to the ability of graphene derivatives to passivate surface/interfacial defects between perovskites and charge transport materials,^109^ and lower defect concentrations by promoting the uniform growth of larger crystals (from 1024 nm up to 1250 nm).^36,107^ Graphene derivatives can participate in defect passivation at both exterior and interior film surfaces through bonding of their functionalities (–NH_2_, –OH, –C=O) to uncoordinated ions on the perovskite surface.^109,110^ Hence, graphene derivatives, such as GO, have lucrative research prospects for defect passivation in PSCs.

The conventional and inverted planar architectures of graphene derivative-based PSCs have achieved PCE values of 18.2 and 13.8%, respectively.^44^ In recent times, other positive prospects have shown the inclusion of NiO–RGO in a glass/FTO/Sr–TiO_2_/Al_2_O_3_–graphene/NiO/perovskite/NiO–RGO/spiro-OMeTAD/Au configuration, achieving 95% of the theoretical photocurrent density of 27.2 mA cm^−2^ (Table 1).^111^ This was driven by the ability of NiO–RGO to lower the trap state concentration of electrons and holes (from 7.09 × 10^15^ to 3.59 × 10^15^ cm^−3^ and 9.57 × 10^16^ to 2.71 × 10^16^ cm^−3^, respectively), and facilitate fast charge mobilities (from 4.12 × 10^−2^ and 1.12 × 10^−1^ cm^2^ V^−1^ s^−1^ to 5.05 × 10^−1^ and 1.33 × 10^−1^ cm^2^ V^−1^ s^−1^, for holes and electrons, respectively). This highlights the suitability and potential benefits of using RGO composites in PSC technology.

Other important aspects of a PSC device are linked to conductivity and band alignment of constituent materials. It is difficult to balance the band alignment effects of GO with conductivity properties towards effective charge separation; hence, more research is needed. While the high work function (WF) effects of graphene derivatives promote better band alignment, ohmic contact and building of a potential difference towards higher *V*_oc_, an increased conductivity leads to a high charge collection efficiency and *J*_sc_.^21,112^ On the one hand, the valence band position of the HTL should align with the HOMO of the perovskite layer (Fig. 1).^56,99^ For example, the synergy between GO and polyvinyl acetate was reported to lower the energy level difference between the valence band of the perovskite and the WF of a carbon electrode (from 0.56 to 0.28 eV), and increase hole extraction (shortened PL lifetime from 0.36 to 0.29 ns) between polyvinyl acetate and carbon in a FTO/compact TiO_2_ (c-TiO_2_)/m-TiO_2_/perovskite/polyvinyl acetate/GO/carbon cell.^13^ On the other hand, the LUMO of the perovskite should align with the LUMO of the ETL (Fig. 1). Theoretically, graphene has a WF of 4.5 eV,^112^ while derivatisation to GO and RGO was associated with WF modulation into the range of 4.35–5.28 eV^46^ and 4.9–5.0 eV,^112–114^ respectively. The WF of GO can also be tailored by heteroatom functionalisation (by replacing oxygen moieties), by reacting GO with molecules such as NH_3_ ^85^ and doping^21,115^ with elements such as B^99^ and F.^114^ For example, silver trifluoromethane sulfonate doped GO achieved a lowered *R*_s_ (from 16.44 to 12.11 Ω cm^2^) and electron/hole recombination due to WF modulation.^116^ Mann *et al.*^117^ also reported RGO WF modulation to 5.34 eV *via* sulfur doping that improved functionality of PEDOT:PSS to achieve a 19% increase in the PCE and improved stability (stable PCE after 10 days for sulfur–RGO/PEDOT:PSS *versus* continuous decline for 30 days in control PEDOT:PSS devices). The graphene derivative dopant must increase performance without compromising other parameters, such as transmittance and charge extraction. For illustration, Kim *et al.*^118^ altered sheet sizes of GO by sulfur-doping, and this influenced the extraction of charge carriers in a PSC (PCE increased with decrease in size of GO with a corresponding increase in PL lifetime: 6.55, 8.87 and 11.3 ns for <1, 1–16 and >25 μm^2^ sizes, respectively), highlighting the importance of optimising sheet sizes. Composite synthesis was also reported to modulate the WF; for example, MoO_3_ and Ag raised the WF of GO from 4.23 to 4.71 eV^119^ and 4.95 eV,^103^ respectively. Another reported functional composite example is GO–Li, with a lowered GO WF of 4.3 eV (from 4.9 eV).^120^ This was rationalised by the ability of Li to donate a valence electron to GO and the associated induction of dipoles by Li^+^; therefore, the Fermi level shifts towards vacuum and the WF is minimised.

GO is transformed to a semiconducting character by tuning the associated WF through reduction.^95,121^ Whilst it is a good fabrication strategy to utilise the high dispersibility of GO in promoting substrate coverage with perovskite, the subsequent reduction process may introduce complications in PSCs. On the other hand, despite aggregation effects and poor dispersion-ability from prior GO reduction, fabrication may require the use of both surfactants (such as sodium dodecylbenzene sulfonate)^22^ and chemical reductants (such as hydrazine hydrate),^22,121^ which may be difficult to handle at large-scale due to their toxic and explosive properties. A moderate annealing temperature in air (usually 200 °C), UV radiation and chemical treatment are possible routes to reduce GO towards improved device conductivity.^24,56,122,123^ Annealing graphene derivatives at high temperatures (above 500 °C) for PSCs is not suitable for most flexible substrates and perovskites (stability and contact issues). Typical contact problems can be improved by using volatile pore-forming agents that evaporate upon thermal treatment, leaving voids occupied by the perovskite.^21^

The effective role of graphene derivatives in PSCs still needs further in-depth understanding. For example, Kim *et al.*^46^ studied the effect of the oxygen content of GO (7, 10 and 18%) used as an additive to the HTL (spiro-OMeTAD) of PSCs and comprehended that 10% oxygen content was the optimum with a negligible drop in PCE after 18 days of storage under a RH of 20%. Cho *et al.*^124^ investigated the most effective location of RGO in a PSC and obtained the best results when it was used as an ETL component (3% better than control, while PCEs of the rest were below that of the control). A similar study reported a 20% PCE improvement through a simultaneous inclusion of RGO in the ETL and active layers of a PSC.^107^ From the WF discussion, it is possible that the oxygen concentration influences RGO performance in PSCs. This view is supported by density functional theory (DFT) calculations that showed that the epoxy oxygen concentration tunes the charge extraction ability as follows: if <33% then both holes and electrons can be extracted, for the 30–66% range electron transport is permissible, but if >60% no extraction is conceivable.^125^ However, since it is rare to find only one particular type of oxygen species in a graphene derivative after synthesis, it is not easy to attain such tailoring due to the presence of other moieties.

Here we detail the suitability of graphene derivatives in PSCs as ETL,^115,126,127^ HTL,^56,81,82,115,126,128^ buffer and active layer additives,^56,81^ and transparent conducting electrodes (TCE) (Table 1).^22,81,122^ The widespread roles of graphene-based derivatives in PSCs affords advantages that most likely arise from favourable interactions with other components, configurations, experimental conditions, unpredictable perovskite nature, and varying the oxygen species and their concentration. However, due to the large volume requirement, the most likely drawbacks are linked to solvents and large-scale GO-based PSC production.^21^

### 3.1 Transparent conducting electrode

The role of a TCE, commonly designed to be the layer facing the light source, is to collect electrons from a semiconductor and transfer them to the external circuit. The most common TCEs are fabricated from indium- and fluorine-doped tin oxide (ITO and FTO). The problems associated with these commonly used TCEs include high fabrication costs (contributes 58–73.9% of the material costs)^7,129^ and energy consumption, natural brittleness, and the scarcity of indium (in the case of ITO glass), which is estimated to be 0.05 ppm and 0.072 ppm in the continental and oceanic crust, respectively.^130^

Theoretical simulations conducted with COMSOL and SCAPS-1D software have predicted graphene-based materials to be effective as either top or bottom TCEs in PSCs as they offer better heat dissipation and stability than their metallic counterparts.^131,132^ The thermal stability of RGO promotes heat dissipation within the PSC,^95^ hence, reducing the risk of thermally-induced device degradation. While the use of pristine graphene as both front and back electrodes can work,^78,133,134^ its intrinsic low wettability, due to its hydrophobic nature, causes the formation of poor perovskite films, limiting its usage. Other graphene derivatives, such as GO and RGO, are suitable PSC TCE alternatives due to their good mechanical strength (Young modulus of 1 TPa), ability to facilitate heat dissipation, and lower perovskite degradation and ion migration rates at elevated temperatures. P-Type graphene derivative-based TCEs, such as GO, are relatively stable in air and therefore easily produced.^135^ GO reduced by chemical or laser means is suitable for TCEs fabricated by spin-coating and result in PSCs with low charge impedances.

Despite the usefulness of graphene-based derivatives as TCEs, their practical application has been greater in other PSC layers, as reviewed in the subsequent sections. This is probably due to limitations in light transmittance to the perovskite layer, because graphene derivatives absorb visible light, and difficulties in deposition onto substrates. The drawbacks of replacing common TCEs in PSCs with solution-processed GO include lower conductivities and PCEs.^95^

Graphene derivatives are appropriate for PSCs that use transparent and flexible substrates such as cellulose and plastics.^21,81^ Examples of graphene derivative-based TCEs in a flexible PSC are single-walled carbon nanotubes (SWCNTs)–RGO,^81^ SnO_2_–GO,^136^ Au–Cl_3_-doped graphene^137^ (with the use of 3-aminopropyl-triethoxy silane as adhesion promoter), and Ag nanowires–GO (Table 1).^122^ Despite the high prospects of Ag nanowires in replacing ITO and FTO due to high transmittance and low sheet resistance, they suffer from high costs and poor chemical stability when exposed to halides. Ag nanowire–GO composites are able to improve the chemical stability in flexible PSCs.^122^ However, the hydrophobic nature of the Ag nanowire–GO composite can also introduce problems in the HTL and perovskite film during fabrication.^122^

Although graphene derivative-based TCEs are more appropriate for inverted PSC types, applications in conventional devices have also been reported. As an illustration, Kim *et al.*^135^ reported a Ag nanowire-doped graphene-based TCE for n–i–p applications that exhibited a PCE of 15.8 and 13.5% for rigid and flexible PSCs, respectively. Hence, this could possibly be extended to GO and RGO in which they can be additional components or substitutes for traditional materials either as transparent front electrodes or electron acceptors.

### 3.2 Active layer

The role of an active layer in a solar cell is to harness sunlight and, in turn, create charge carriers. Several graphene derivative-perovskite composites for use in the PSC active layer have been reported for a variety of reasons (Table 1). For instance, graphene derivatives such as N–RGO-perovskite composites have two possible effects on PSCs; firstly, by increasing the grain size through a decelerated crystallisation step, both the *J*_sc_ and FF are increased,^138^ and, secondly, the composites retard the rate of recombination, thereby increasing the *V*_oc_. Other typical examples are the GO-modified perovskite materials reported as sensitizers that improved hole mobility due to the enhanced perovskite quality arising from a smaller number of grain boundaries (grain size increased from 150 to 200 nm) and improved uniformity.^29^ The optimum performance of the composites was subject to the wt% of GO (optimum = 0.5 wt%). This means that even if the inclusion of graphene derivatives has benefits, maximum gains to overall PSC performance require optimisation of the amounts of components to avoid the formation of inhomogeneous films that culminate in a disrupted crystal quality. In summary, solution-processed graphene derivatives have the potential to improve crystallisation, morphology and charge transport in PSCs.

### 3.3 Blocking layer

Buffering in PSCs is important in several ways; for example, in minimisation of the reaction between extracted charges and X^−^.^110^ Buffering layers are utilised in blocking holes and electrons from collecting at the anode and cathode, respectively. This ensures a balanced charge extraction towards the appropriate electrodes.^81^ Common buffering layers in PSCs are TiO_*x*_ and PEDOT:PSS. Although the PSS component of PEDOT:PSS lowers the overall conductivity,^139^ its hygroscopic nature is manipulated for dispersion in water as a counter ion.^101^ The acidic and hygroscopic nature of PEDOT:PSS,^101,140^ as well as moisture sensitivity of TiO_*x*_, are some of the common setbacks of current blocking materials. Moisture in PSCs will not only deteriorate the perovskite at atmospheric conditions but also reduce electrode conductivity.^141^ Additionally, PEDOT:PSS has an intrinsic inefficient electron blocking ability.^36^

GO generates structural inhomogeneities through its high oxygen functional group concentrations that can aid retardation of electron/hole recombination by effecting low electron mobility rates.^140,142^ For example, the wider band gap of GO induces an effective blocking layer in PEDOT:PSS–GO composites (Table 1).^56^ The PEDOT:PSS–GO composite is currently among the most effective materials that can act as an electron blocking layer and HTL between the active layer and electrodes in order to minimise current leakage and electron recombination. Although improving the PCE with composites of graphene derivatives for use as HTLs and ETLs is gaining momentum, their use as a blocking layer is still less common than their TCE applications.^143^ Attributes of graphene derivatives as buffer materials include solution processability, excellent stability, low fabrication costs and their diverse WF tunability. The WF modulation of GO through reduction is a feasible way to engineer energy barriers between the HTL and active layer.^143^ Heat and moisture barriers between the perovskite and the electrodes in a PSC can be created by inserting graphene derivatives.^14^

Other graphene derivatisation examples for blocking purposes include SWCNTs- and amine-modified GO (Table 1).^144^ A fluorinated (–CF_3_)–GO was reported to promote both oxidation resistance and moisture adsorption, thereby inducing better device stability (Table 1).^51^ Another example is a poly(methyl methacrylate) (PMMA)–GO composite buffer that was reported to block electrons and increase carrier transport selectivity through shunt resistance (*R*_sh_) enhancement and a decrease in *R*_s_, respectively.^145^ Also, the strong chemical interactions between NiO, RGO and the perovskite in a NiO–RGO blocking layer were envisaged to boost stability against reactions with air and water (Table 1).^111^

### 3.4 Hole transport layer

The role of HTLs is to extract holes generated in the perovskite layer.^146^ In some instances, PSCs have been reported to function without a HTL but with just an insulator that separates the two electrodes.^21,147–149^ In such cases, the perovskites will act as a sensitizer and an ambipolar charge transporter.^138,150,151^ Exclusion of a HTL is beneficial as it can reduce material use and fabrication costs and aids in resolving stability issues such as those from increased moisture effects that may be introduced by hygroscopic HTLs such as PSS (a component of PEDOT:PSS). However, the HTL can improve the PCE by reducing recombination rates and enhancing *V*_oc_. The *V*_oc_ in PSCs is mostly influenced by recombination at interfaces. This effect is large in HTL-free devices due to the longer-lived duration of charges at interfaces. Another driver for HTL use is that the surface morphology of the HTL positively influences electrical properties such as *R*_s_ and *R*_sh_. This is important because a large *R*_sh_ infers small charge carrier recombination in the active layer and high selectivity of charge collection.^101,152^ An efficient hole selective collection will occur when current leakage in the reverse bias is decreased.^152^

To be a suitable HTL in PSCs, the material requires an appropriate energy level, low deep-trap state density, sufficient charge extraction rate and transfer capabilities.^83^ A commonly used HTL for the p–i–n configuration is PEDOT:PSS due to its tuneable conductivity and high optical transmittance within the visible region.^82,115^ Also, since the WF of GO (4.9 eV) and that of PEDOT:PSS (5.1 eV) align well, GO is able to decrease the associated *R*_s_.^56,140^ Copper phthalocyanine^27^ and spiro-OMeTAD^142,145,148^ are also well reported functional HTLs. Owing to a compatible energy level with perovskites, spiro-OMeTAD is common in Au-based PSCs though it requires a complicated multi-step synthesis, is expensive and associated with low intrinsic charge carrier mobility, and thus requires dopants to perform well.^12,15,21,54^

GO functions as a hole acceptor and transporter,^80,153^ but has a lower conductivity than RGO, rendering it an inferior transporter.^154^ The GO HTL can tailor optical properties,^155^ crystallisation and morphology of perovskites.^140^ A uniform, compact and void-free GO layer is important for device performance, but some studies have encountered difficulties in achieving this with GO.^56,115^ Unlike N–RGO, RGO has been reported in some instances to cause deterioration of perovskite morphology.^95^ Cracks and voids in the perovskite layer are recombination centres with poor uniformity leading to increased shunt paths, current leakages, and low voltage.^21,115,156^ This phenomenon is subject to the deposition method and is a signal of the importance of uniform layers throughout a PSC. Furthermore, inhomogeneity of GO layers on TCEs produces non-uniform electrical characteristics due to either direct contact between the perovskite and TCE or insulating behaviour that results in poor hole transport.

Lee *et al.*^56^ overcame this shortfall by a repeated spin-coating of GO and PEDOT:PSS to reduce surface roughness. Sequential solution deposition has achieved decent, repeatable, and easily controlled deposition, proving better than both vacuum deposition and vapour-assisted solution-processing.^142,157^ Nouri *et al.*^115^ achieved a uniform thin film by first cleaning FTO with ethanol to increase its hydrophilicity, and then spin-coating twice with GO dispersed in isopropanol at 200 rpm (PCE increased from 4.4 to 10.2%). Feng *et al.*^85^ reported PEDOT:PSS/GO–NH_3_ as a HTL with improved optical transparency, texture and complete substrate coverage morphology. NH_3_ treatment of GO used as a HTL was also reported to increase the PCE of GO-based inverted PSCs, by 12%^85^ and 18%,^158^ and the PSC device was more stable than that from pristine GO due to advanced *V*_oc_ and FF, and preferred crystal orientations, respectively (Table 1). The enhancement was ascribed to a decrease in current leakage and increased *V*_oc_ from the compact perovskite morphology induced by NH_3_–GO. Selvakumar *et al.*^159^ reported an increase of both mobilities (from 0.595 to 4.850 cm^2^ V^−1^ s^−1^) and *R*_sh_ (from 152 to 10 669 Ω cm^−2^) but a decrease in conductivity (from 4820 to 1521 S cm^−1^) upon B-doping of GO. Habib *et al.*^160^ reported an optimised ratio of PANI : GO of 1 : 0.5 with a sheet resistance of 0.067 Ω cm^−2^ and PCE boost of 125% due to reduction of current leakage from smooth surfaces (root mean square of 12.65 nm) in the bulky materials.

The lifetime of a PSC is subject to pH, humidity level, permeability, density and hydrophobicity of the HTL.^38,161^ This means that PEDOT:PSS limits lifetime and cell performance due to its hygroscopic nature.^101^ Encapsulation improves device stability and can maintain the integrity of PSC layers over time.^74^ The use of graphene derivatives as encapsulant materials has the potential to contribute significantly to enhancing perovskite device longevity. An illustration of this is the reported lowering of the hygroscopic character of PEDOT:PSS through the synthesis of an annealed composite with GO.^162^ However, depending on their chemical nature, graphene derivatives may pose drawbacks in their long-term utilisation as HTLs since they may introduce considerable moisture in the active layer. This is detrimental to PSC devices because moisture dissolves the methylamine and/or hydrates the perovskite and causes it to decompose to PbI_2_ in a few hours or days when exposed to air and moisture.^38,85,101^ Possible ways to keep moisture out of the perovskite layer and thereby minimise perovskite degradation include employing a hydrophobic HTL such as hydrophobic RGO. Another graphene derivative-based HTL with minimal hydrophilicity is thiolated GO as in the perthiolated trisulfur-annulated hexa-*peri*-hexabenzocoronene–GO composite form.^83^ Additionally, a defect-free monolayer of some graphene derivatives is impermeable to all gases, preventing O_2_ diffusion into the perovskite.^163^ For instance, F–RGO HTL was able to minimise moisture and O_2_ attack on the perovskite layer resulting in 72% PCE retention after 30 days (the control PEDOT:PSS-based device malfunctioned in 9 days).^146^ Similar reports detail an isopropanol ultrasonically-treated graphene-based encapsulant enhancing the stability of a PSC by 90% within 60 h by reducing oxygen oxidation and moisture attack.^164^ In addition, the high thermal conductivity of graphene helps with thermal stress on PSCs, leading to additional stability benefits.^95,109^ A graphene derivative–carbon paste has been reported as a suitable encapsulant of PSC modules of areas 0.09, 1 and 4 cm^2^ that achieved champion PCEs of 15.81%, 14.06% and 13.86%, respectively.^165^ Additionally, graphene-based encapsulants are compatible with roll-to-roll fabrication through a dry transfer method that has attained PCE retention of 82.4% after aging in ambient air for 3700 h.^163^ This demonstrates that graphene derivatives, as encapsulants, are beneficial and conducive to the future scaling-up of PSCs. Other hydrophobic materials, such as a cross-linked compacted poly(methyl)methacrylate (PMMA) layer on top of the HTL,^145^ have been used to modify the perovskite surface and seal the active layer from atmospheric effects and therefore increase stability. The compatibility of graphene derivatives with common PSC encapsulants, such as PMMA, has been illustrated. For example, a PSC encapsulated with a PMMA–RGO composite displayed excellent performance under operational conditions because the OH^−^ moieties on RGO interact with the C=O on PMMA.^95,109^ However, strict and effective encapsulation may introduce not only additional cost (60% more)^38,166^ but also hinder optical transparency.^167^ Hence, a careful cost-effective sealing innovation needs to be sought for moisture insensitive designs involving graphene derivative HTLs. In a study to compare stability and PCE upon inclusion of GO in PSC devices, the glass/FTO/NiO/GO/perovskite/GO/TiO_*x*_/Al cell structure was more stable but with a 25% lower PCE than the glass/FTO/PEDOT:PSS/MAPbI_3−*x*_Cl_*x*_/PCBM/Al configuration.^168^ Hence, further understanding of the degradation mechanism through focused studies on individual PSC layers rather than the overall PSC device may provide solutions to both stability and PCE enhancement.^169^

For HTL purposes, graphene can be chemically modified as an interlayer material to simplify the electrode solution coating process by improving wettability during the coating process. The enhanced wettability from derivatized graphene reduces the nucleation barrier to form a better quality perovskite layer without pinholes.^101^ The perovskite quality boost occurs *via* heterogeneous nucleation (nucleation at foreign nuclei or surface), and this process is influenced by the surface or interface contact energy (surface chemistry and morphology effects).^170^ This infers that improved wettability (small contact angle) is associated with a small contact energy barrier and, together with a uniform large surface area, promotes continuous crystal growth. For illustration, GO was modified with 4-fluoro phenyl-hydrazine hydrochloride (4FPH) to form a 4FPH–GO composite with increased passivation of trap states close to the perovskite surface (disorder parameter that represents broadness of trap state densities decreased from 151 to 81 meV) and improved charge diffusion length (from 400 to 700 nm) for effective hole collection, and hydrophilicity for easy coating (Table 1).^152^ Furthermore, the use of GO has been shown to decrease the contact angle between spiro-OMeTAD and the perovskite (from 21.6 to 2.5° ^101^ and from 13.5 to 0° ^142^), leading to better spreading of the HTL on the perovskite.

HTL surface properties, structure and optical characteristics also influence film quality and suitability in effective light-harnessing. The optical properties of a HTL are important because it is the first component to significantly interact with light in the inverted configuration.^80^ Hence, GO absorption of 2.3% of incident light in the 200–800 nm range may affect the overall PCE.^26^ Hence, a high concentration of graphene derivative additives introduced into the HTL and poor dispersion can cause reduced film quality (light transmittance) and ultimately lower PCEs.^21,80^ This aspect is closely related to the film thickness effect as reported with functionalised GO and composites.^36,135,152,153^ Despite the possible increase of *R*_s_ due to the high oxygen concentration in GO,^101^ the quenching effect has also been reported to increase with composition.^25^ For illustration, a 6.7% PCE was reported for a single GO layer (80% quenching effect improvement) in a PSC device, whilst a bilayer recorded 11.5% (91% quenching effect boost) due to the formation of an effective Schottky barrier, minimising recombination effects.^171^ In brief, there is a trade-off between optical transmittance and electrical resistance. On the one side, the electrical resistance is lowered by thick graphene derivative sheets, whilst on the other side, thin films have higher optical transmittance.^22,120,140,143^ Resistivity of graphene was shown to decrease from 1050 to 140 Ω sq^−1^ when the thickness was increased from 1 to 2 layers.^127^ SCAPS-1D simulations have also shown the effect of thickness on *V*_oc_ up to 1 V and an optimum thickness of 30 nm, and a decline in FF with thickness increase.^132^ Since several works have reported *V*_oc_ above 1 V, this effect is likely to be dependent on the material properties (Table 1).

To sum up, the review of graphene derivatives in the context of HTLs shows their potential in increasing hole transport and their electron blocking effect, thereby improving the electrical properties of the device (conductivity and *R*_sh_), FF and *V*_oc_.^36^ Additionally, graphene derivatives have generally been able to improve the perovskite crystallisation process producing larger crystals, TCE surface coverage and in-plane orientation towards effective hole extraction.^25,101^

### 3.5 Electron transport layer

The function of an ETL in a PSC is to extract and transport generated electrons away from the perovskite active layer. The three common classes of ETLs for PSCs are metal oxides, n-type conjugated polymers, and fullerenes.^115^ A typical example of fullerenes for ETL is phenyl-C_61_-butyric acid methyl ester (PCBM). PCBM facilitates a fast charge transfer from the perovskite (approx. 0.4 ns), and its hydrophobic nature enhances PSC stability.^140^ Due to the low cost of graphene derivatives and their ability to introduce additional percolation channels for electron transport, solution-processed graphene derivatives have the potential to advantageously replace the popular PCBM.^56,81^ Hence, the basis of adding graphene derivatives to the active layer as ternary components of polymers and fullerenes is to ultimately increase carrier mobility.

Although a report indicates that PSCs can also function without an ETL (with PCE of 10%),^172^ ETLs are important in lowering interfacial energy barriers between electrodes and the perovskite active layer. Hence, electron/hole recombination rates are lowered by the ETL through electron extraction and transfer enhancement.^173^ The tuning of the ETL surface energy is a feasible control protocol for wettability, recrystallization and nano-structural parameters of perovskites.^173^ With reference to the PSC structure, the n–i–p type is largely influenced by the electrical, optical and structural features of an ETL.^35^ Common ETLs for the n–i–p structure are oxides, such as TiO_2_,^34^ SnO_2_ and ZnO.^35,44,174^

The processing temperature of an ETL during PSC fabrication influences device performance. For instance, both c- and m-TiO_2_ require a high sintering temperature (*ca.* 450 °C) for optimum crystallinity and charge mobility.^21,37,175^ A high sintering temperature hinders applicability in widespread areas, such as wearable and flexible electronics. ZnO is a good alternative because it requires a low sintering temperature and time; however, its non-optimised dimensions (diameter: ∼80 nm and length: ∼1 μm) negatively affect perovskite crystal growth in PSCs.^77^ A m-TiO_2_ layer may hinder electron transport due to roughened grain boundary scattering.^26,176^ Ternary n-type semiconducting metal oxides with similar characteristics as TiO_2_, such as high Hall electron mobility and small effective mass, are also effective ETLs that have the potential to replace TiO_2_ in PSCs.^153^ For instance, the TiO_2_ hysteresis shortfall has been resolved by the use of SnO_2_ despite its low thermal stability, and non-optimal optical and electrical properties.^35,84^

Interestingly, graphene derivatives are compatible with metal oxides that function in ETLs to form meso-super-structured PSCs with preferred properties (Table 1),^25^ such as increased semiconducting properties,^121,173^ device stability and charge extraction.^27^ For illustration, the inclusion of RGO within TiO_2_ layers of PSCs produced a PCE boost of 21%.^27^ A typical example is the overturning of the slow electron diffusion rate limitation in TiO_2_, due to high resistance and low charge collection efficiency, by the use of GO as an interfacial layer between the TCE and TiO_2_.^26,34,37,124^ Although all values were lower than the FTO control (*J*_sc_ of 17.49 mA cm^−2^ and FF of 63%), altering the % of GO in c-TiO_2_ has also been reported to proportionally enhance *J*_sc_ and FF due to increased electron transport through the creation of thermodynamically favourable energy paths and lowered recombination.^22^ Xie *et al.*^177^ reported a *V*_oc_ boost of up to 1.12 V through RGO inclusion in a MoO_3_ ETL (Table 1). Additionally, N–GO was used to reduce oxygen vacancies in SnO_2_ ETLs as a control measure (5 volume% lowered O 1s peak in X-ray photoelectron spectrum and improved PCE by 8%, Table 1).^35^ In another study, a RGO/Zn_2_SnO_4_ nanofiber scaffold was reported to effectively reduce recombination relative to pristine Zn_2_SnO_4_ (slow PL lifetime was reduced from ∼17 to 4 ns, Table 1).^153^ Also, ZnO–RGO quantum dots were reported as a PSC ETL, and the RGO passivated the ZnO effectively, improving charge carrier extraction and the stability of the perovskite due to a decline of the OH^−^ concentration on the surface (10% PCE drop *versus* 90% of control after one month).^178^ In another example, Li neutralised GO was reported to increase electron extraction from the perovskite as an ETL.^120^ In short, the electron conductivity of an ETL plays an important role towards effective charge extraction from active layers in PSCs, and compositional effects must be investigated in each case when graphene derivatives are involved.

### 3.6 Tandem-structured devices

**Fig. 4:**
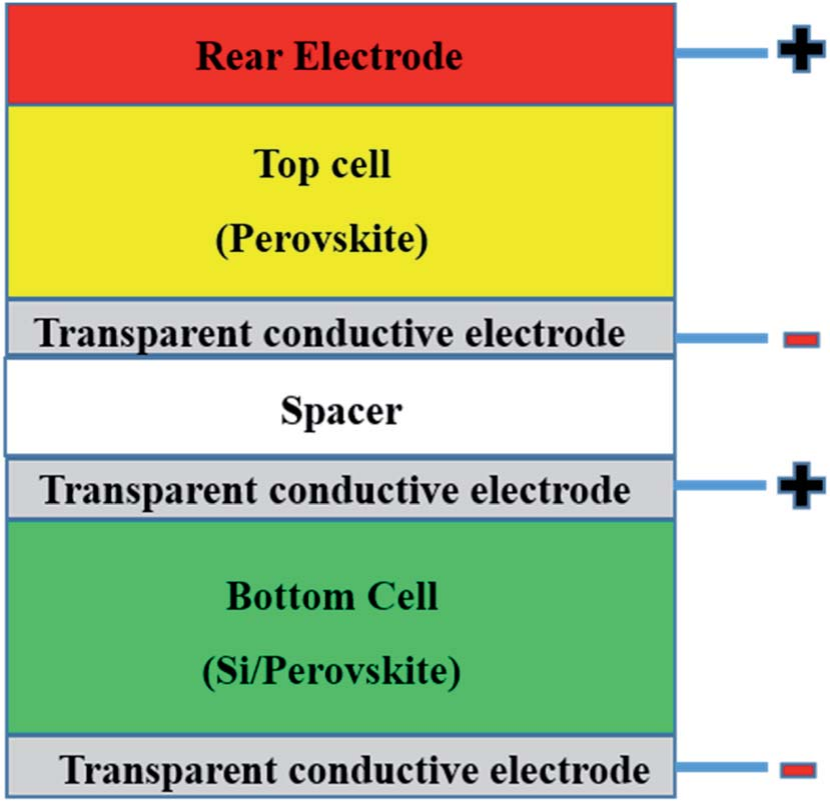
Representative tandem structure involving PSCs in a four-terminal cell.

Tandem solar cells (TSCs) are stacked narrow band gap and wide band gap subcells with high potential to suppress the Shockley–Queisser limitation of single solar cells and increase *V*_oc_ and *J*_sc_ (Fig. 4).^165,179,180^ This means only photons with energy > band gap of a semiconductor are absorbed and not all absorbed photons can be converted to electricity due to thermalisation of charge carriers.^181^ Leading tandem structures involving perovskites are perovskite/perovskite (17% share), perovskite/CIGS (25% share) and perovskite/Si (58% share).^179^ Perovskite/Si TSCs have reached a certified value of 29.5%.^33^ Illustratively, graphene has been shown as a suitable material for TCEs (PCE of 8.3%),^182^ electrodes (PCE of 18.1%),^183^ and as an additive to the HTL (PCE of 6.02%)^182^ of TSCs. Graphene derivatives can hypothetically slow down charge thermalisation processes.^165^ However, there are currently scarce applications of graphene derivatives in TSCs. Theoretically, since a tandem structure will require an efficient PSC, the demonstrated positive effects of graphene derivatives in various layers of PSCs (Table 1) infers that graphene derivatives are promising in TSCs. Hence, the progress of integrating graphene derivatives in TSCs is dependent on their status in PSCs. Since TSCs require highly conductive intermediate layers with low visible light absorption capabilities to separate subcells,^127,182–184^ the future application of graphene derivatives in TSCs must pay much attention to conductivity and transparency dynamics.

## 4. Deposition techniques for graphene derivatives and stability of PSCs

Most graphene derivatives utilised in PSC fabrication, as summarised in Table 1, are synthesised by means of exfoliation through chemical oxidation of graphite. The oxide form of graphene can easily be processed from solution. Deposition protocols for graphene derivatives in PSCs need to be performed in a way that incurs minimum damage to other underlying layers to preserve the integrity of the materials and prevent defect generation and promote scaling-up.^114^ The PSC fabrication methods involving graphene derivatives include electro-spraying,^152^ radio-frequency magnetron sputtering,^128^ screen-printing,^185^ lamination,^119^ doctor blade (blade coating)^21^ and spin-coating^82^ (Table 1). The spin-coating conditions (speed/time, annealing temperature, solvent) should be quantitatively managed to control morphology and thickness (thicker layers will be less transparent).^17,24,54,156,186^ Yang *et al.*^54^ suggested that a perovskite thickness of at least 300 nm is sufficient for meaningful light absorption. The spin-coating method is the most popular and conventionally used in the fabrication of lab-scale devices.^187^ However, large-scale manufacturing through spin-coating may produce pinholes, rough perovskite films and non-uniform coatings;^30,87^ hence, deposition studies still require more insights to avoid PCE losses during scale-up. Solution-coating processes support scaling-up, and typical methods include roll-to-roll,^188,189^ and thermal- and air-assisted blade coating at ambient conditions.^113^ Blade coating involves a solution/paste being dropped on a substrate followed by sweeping across the substrate with a blade-like implement.^113^ Graphene derivatives are compatible with scaling-up deposition methods as illustrated by a K–GO, ETL/perovskite interlayer, coated by means of a blade-slot die (area increase from 0.1 to 16 cm^2^ slightly decreased PCE from 18.3% to 16.1%).^187^ The roll-to-roll method has been successfully employed for PSCs with Li–GO as a TiO_2_/perovskite interfacial material for a module with an area of 50.6 cm^2^ and PCE of 12.6% (relative to 13.5% for an area of 0.01 cm^2^).^30^ Screen-printing is another scaling-up technique in which the paste composition and conditions can tune properties.^190^ The technique is suitable for scaling-up thin film deposition, usually at a speed greater than 6 m min^−1^ but with long drying and sintering durations at high temperatures for better porosity.^191^ An investigation of the use of near-infrared heating was reported to substantially reduce the processing time at a speed of 2 m min^−1^.^191^ The ink of graphene derivatives can be cheaply produced in high yields using non-toxic solvents to support integration into large PSC modules with competitive PCE.^30^ Transfer of graphene derivatives through polymer carriers has led to performance deterioration; hence, it is difficult to fabricate effective TCEs.^110,192^ Despite knowledge gaps, graphene derivatives, particularly the oxide forms, are compatible with flexible substrates because of their high dispersion in solvents and the ease with which they can be coated; hence, future research directions can explore their use in improving the cost, stability, and performance of PSCs.

A fundamental aspect in PSC technology yet to be overcome is stability with respect to humidity (Table 1). Reference to the RH is necessary whenever investigations are done to ascertain the suitability of materials at different locations.^76^ An analysis of PSC stability in terms of the decline in PCE upon graphene derivative inclusion as a p–i–n HTL component, solely a p–i–n HTL, the hole blocking layer component, active layer constituent, and ETL n–i–p constituent/blocking layer, after roughly 30 days show PCE declines of 12, 10, 50, 30 and 35%, respectively (Table 1). This may infer that graphene derivatives provide the greatest stability boost in p–i–n HTLs and the worst stability in p–i–n hole blocking layers. From the reviewed articles, the application of graphene derivatives in conventional PSCs has recorded the highest values of *V*_oc_, *J*_sc_ and FF of >1 V, 26.96 mA cm^−2^ and 82.80%, respectively (Table 1). Interestingly, the highest PCE boost of 21% was with graphene derivatives mixed with the perovskite layer.

## 5. Prospects and challenges

From the golden triangle (cost, PCE and stability) perspective,^60,166^ PSCs are promising for commercialisation due to both a fast-growing PCE and low fabrication costs. However, there is still a need to improve the stability and sustainability of devices, such as removing the use of toxic solvents in manufacturing^43^ and mitigating the use of toxic lead through substitution or, failing this, sequestration and accountability throughout the lifecycle of devices. From the current review, it is clear that substantial studies have been dedicated to PSCs as an emerging technology, including optimisation of perovskite crystallisation, interfacial engineering and flexible substrate developments. The desired characteristics for PSC fabrication are homogeneous and continuous highly crystalline large perovskite grains, with high stability to moisture and atmospheric conditions. Since it may be challenging to develop a graphene derivative that addresses all shortfalls of PSCs and because some focused studies have shown their partial potential support to, particularly, performance and stability, future research should focus on the synergy between factors.

From the reviewed articles (Table 1), it is clear that the addition of graphene derivatives to PSC layers inhibits degradation of perovskite through defect passivation (promote uniform deposition to eliminate pinholes and promote perovskite growth), thus improving stability. This is because degradation is initiated at grain boundaries and defective regions.^187^ Reduced defect intensity in perovskites and interface passivation induced by graphene derivatives also lessens charge traps; therefore, promoting performance.^60,95^ Regulation of perovskite crystal growth by using graphene derivatives improves reproducibility of deposition of the active layer.^165^ Graphene derivatives in PSC charge carriers (by virtue of demonstrated tuneable physicochemical properties *via* morphological and chemical transformations) promote performance by reducing electron/hole recombination.^30,221,222^ This is achieved through WF and band gap modulation that create appropriate energy level alignment between perovskite and charge carriers (ideal *V*_oc_). Graphene derivatives also generate high *J*_sc_ and FF *via* interfacial chemical modifications that promote high electron extraction and transport from the ETL due to relatively higher conductivity. Additionally, graphene derivatives as dopants to the ETL suppress octahedral tilt in the perovskite monolayer to create nanoscale ferritic distortion with irreversible polarisation, thereby increasing electron extraction from the perovskite and minimising electron/hole recombination.^30^

Future use of graphene derivatives in TCEs, though currently less common than pristine graphene, is motivated by the low-cost processing, controllable work function, and resistance to chemical degradation. The hydrophilicity of graphene derivatives has positive prospects for easy dispersions in polar solvents, making them applicable to low-temperature solution processing. RGO is a better option than pristine GO for PSC applications because it is associated with a smaller energy mismatch to the perovskite; hence, promoting electron extraction. The inclusion of graphene derivatives in PSCs needs critical optimisation since it may enhance other relevant parameters at the expense of PCE, as summarised below. For example, graphene oxide (GO and RGO) applications in PSCs still need a conductivity boost through doping or composite synthesis, development of practical, simplified large-scale synthesis, improved film transfer protocols, and optimisation insights towards thin and uniform materials. The film thicknesses of both perovskite and graphene derivatives are crucial factors that also influence the PCE. It is critical to note two contradicting phenomena discussed earlier; firstly, the need for high hydrophilicity during fabrication and sacrificial reaction centre/blocking purposes; secondly, the hydrophobicity requirement in the perovskite for long-term stability and functionality of PSCs. Also, on the one hand, GO-based composites have been reported in some studies to disrupt the homogeneity of spiro-OMeTAD, thereby causing a detrimental effect on PSCs by creating shunt pathways.^124^ On the other hand, composites of GO, such as GO–Li, have also been reported to reduce moisture attack and enhance both stability and life-span through passivation of titania oxygen vacancies by acting as reactive centres and blocking channels to moisture attack.^110,120^ Hence, the current review suggests the need to monitor and balance the basic necessities, particularly cost and performance, of a functional PSC device.

Graphene derivatives are promising alternatives that can positively impact the costs involved in large-scale PSC manufacturing. Relevant current research gaps that involve the use of graphene derivatives in PSCs towards scalable, low temperature processed PSCs include:^21,78^

(i) Improved mechanical infiltration of perovskite solution into graphene derivatives within short intervals and with better reproducibility.

(ii) Attainment of long-term stability under light exposure. Charge transport material should not cause photocatalytic degradation upon light exposure.

(iii) Increasing the temperature tolerance.

(iv) Enlargement of the active cell area.

Photo-induced decomposition is still challenging in PSCs because the perovskite/light interaction is strong; hence, future guidelines include using functionalised additives that also improve chemical interactions between ions.^63^ Graphene derivatives have demonstrated, through an in-depth PSC degradation profile analysis under operational conditions (60% PCE retention (15.8%) relative to control PCE retention of 25% (13.6%) after 24 h light soaking),^61^ to improve long-term stability. This was attributed to a reduction in localised inhomogeneous light-induced decomposition of perovskite back to precursors, preventing I_2_-induced m-TiO_2_ degradation and inhibited diffusion of I_2_ and Au across the whole device.

The use of graphene derivatives as PSC encapsulant materials is an underexplored area;^109^ hence, as a future research direction, focus on in-depth degradation studies of typical devices is essential. Graphene-based materials are good candidates for PSC encapsulants due to their commendable optical properties.^135,163,183^ For instance, 60 μm of a GO encapsulant of a AgNW TCE achieved 83.8% transmittance at 550 nm (preserving 98.4% of the average transmittance of the pristine AgNW TCE).^122^ However, increasing the RGO loading can be detrimental as shown by Nouri *et al.*,^27^ RGO loadings of 0, 0.05 and 0.8 wt% in the ETL achieved PCEs of 13.1%, 13.5% and 12,1%, respectively, due to reduced transparency. Hence, since charge carriers and encapsulants should permit sufficient light to reach the active layer, the future use of graphene derivatives in PSCs should critically investigate concentration dynamics. Also supporting this strategy, is the proven ability of small amounts of graphene derivatives in boosting PCE without altering optical properties.^223^ The realisation of suitable encapsulation materials and protocols prospectively supports the drive to achieve the approximated low 3.5 US cents per kW h target, which is competitive with current commercial solar cells and fossil energy resources upon scaling-up.^165^

From the summarised past research, the attainability of the next stride of PSCs in commercial markets is highly possible if long-term stability, defect concentration minimisation, scalability and processability goals are met. This can be achieved through studies that provide a fundamental and thorough understanding of the effect of graphene derivatives on interfacial parameters and perovskite crystallisation. The modulation of the WF of graphene derivatives has also shown essential benefits in the functioning of PSCs. The current literature has presented a vast number of positive attributes linked with the inclusion of graphene derivatives in PSCs, but further in-depth studies and industrial up-scaling are required.

## Conflicts of interest

There are no conflicts to declare.

## Supplementary Material
